# Exploring the Potential of Thymoquinone-Stabilized Selenium Nanoparticles: In HEC1B Endometrial Cancer Cells Revealing Enhanced Anticancer Efficacy

**DOI:** 10.1021/acsomega.3c06028

**Published:** 2023-10-16

**Authors:** Gonca Gulbay, Mucahit Secme, Hasan Ilhan

**Affiliations:** † Department of Medical Biology, Faculty of Medicine, Ordu University, Ordu 52200, Turkey; ‡ Department of Chemistry, Faculty of Science, Ordu University, Ordu 52200, Turkey

## Abstract

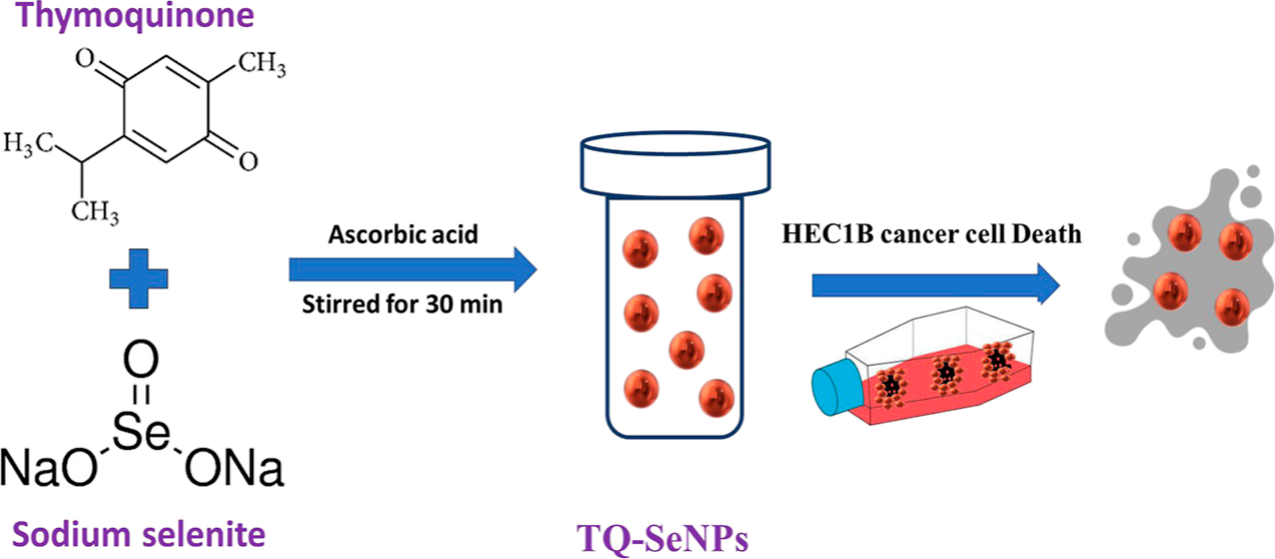

The aim of this research is to examine the potential anticancer properties of thymoquinone (TQ)-encapsulated selenium nanoparticles (TQ-SeNPs) in HEC1B endometrial carcinoma cells. TQ-SeNPs were synthesized, and their size, morphology, and elemental analysis were characterized. Morphological changes were examined by using scanning electron microscopy (SEM). The cytotoxicity and viability of nanothymoquinone were assessed by the XTT (2,3-bis (2-methoxy-4-nitro-5-sulfophenyl)-2*H*-tetrazolium-5 carboxanilide) assay. Gene expressions and protein levels of the mitogen-activated protein kinase (MAPK) signaling pathway were analyzed by real-time PCR and enzyme-linked immunosorbent assay (ELISA), respectively. The decrease in the viability of HEC1B endometrial carcinoma cells was observed in a time- and dose-dependent manner. HEC-1B cells were treated with TQ-SeNP at 40–640 μg/mL concentrations and time intervals, and their viability was assessed by XTT assay. IC_50_ doses of TQ-SeNP in HEC1B cells were detected as 526.45 μg/mL at 48th hour. ELISA indicated that TQ-SeNP treatment reduced the level of p38 MAPK. ERK2, MEK2, and NFKB (p65) mRNA expressions were decreased in the dose group administered TQ-SeNP at the 48th hour compared to that in the control group. However, it was not significant. The novel nanoparticle showed an antiproliferative effect in endometrial cancer cells. However, further studies are needed to increase the anticancer activity of the cell in the TQ-SeNP interaction.

## 1 Introduction

Cancer is one of the leading causes of death worldwide. It is estimated that by 2030, the death rate from cancer will reach 13 million per year.^1^ The basis of carcinogenesis is the gradual combination of mutations that affect biological events such as cell survival, growth control, and differentiation. For this reason, the first affected mechanism in cancers is usually the signal transduction pathways inside the cell.^2^

Inflammation is an important factor in carcinogenesis. Some signaling pathways are activated in response to inflammation.^3^ One of these signaling pathways is the mitogen-activated protein kinase (MAPK) signaling pathway. The MAPK signaling pathway is divided into three major categories: extracellular signal-regulated kinase (ERK), c-Jun N-terminal kinase (JNK), and the p38 kinase pathway. MAPK pathways play critical roles in the development and progression of various human cancers.^4^ MAPKs are key regulators of embryogenesis, cell differentiation, proliferation, and apoptosis. Therefore, members of the pathway are targets of new antineoplastic drugs.^5^

Endometrial cancer (EC) is one of the three major malignant tumors in gynecology, accounting for approximately 30% of malignant tumors of the female reproductive system. Approximately 90% of ECs are sporadic and 10% are hereditary.^6^ Despite advances in the diagnosis and treatment of EC, the incidence of EC continues to rise.^7^ Today, chemotherapy is one of the most effective treatments for endometrial malignancies, as with most types of cancer. However, as with all malignancies, the side effects of chemotherapy that reduce the quality of life still remain. Therefore, herbal studies have been conducted for many years to find new and more effective treatments for malignant cells with minimal risk of toxicity to healthy cells.^8^

*Nigella sativa* is a plant with small black seeds belonging to the Ranunculaceae family. It is often grown in Asian and Mediterranean countries and is widely used in traditional medicine.^9−11^ The seeds of this plant and their essential oils are the bioactive component of thymoquinone (TQ) (2-isopropyl-5-methylbenzo-1, 4-quinone, MW = 164.2).^10^ A wide variety of pharmacological activities of TQ such as anti-inflammatory, immunostimulant, antioxidant, antimicrobial, antidiabetic, hepatoprotective, and cardioprotective have been reported.^12−18^ However, chemotherapeutic and chemopreventive effects of TQ in cancer have also been reported.^19^ In different preclinical studies, it has been shown that TQ plays a beneficial role in the control of inflammatory diseases due to its strong antioxidant activity.^10^ Apart from these, various nanoparticles have been used to increase the bioavailability capacity.^10,20,21^ The rapid development of nanotechnology has led to the emergence of viable new nanomaterials. Nanomaterials such as nanofibers and nanoparticles are characterized by their low weight and high surface-to-volume ratio.^22,23^

The aim of this study was to formulate and characterize TQ-loaded selenium nanoparticles (TQ-SeNPs) and their cytotoxicity in human HEC1B endometrial carcinoma cells.

## 2 Materials and Methods

### 2.1 Chemicals

Thymoquinone (TQ, Sigma-Aldrich, USA), Tween 80 (TW-80), sodium selenite (Na_2_SeO_3_), ascorbic acid, formaldehyde, Triton X-100, and dimethyl sulfoxide (DMSO) were purchased from Sigma-Aldrich (USA). Other reagents used were of analytical grade.

### 2.2 Synthesis of TQ-SeNP

The preparation of TQ-SeNPs involved a redox reaction utilizing sodium selenite and ascorbic acid, following a previously reported method with certain adjustments.^24−26^ Initially, varying concentrations of TQ were dissolved in distilled water (25 mg/mL). Subsequently, 0.3 mL of 0.1 mol/L Na_2_SeO_3_ was combined with different volumes of TW-80 (2 mg/mL), and the mixture was stirred at room temperature for 30 min.

**Scheme 1:**
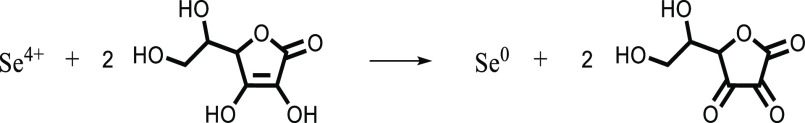
Mechanism of SeNP Synthesis

Following this, a fresh solution of 0.1 mol/L ascorbic acid (1.2 mL) was slowly introduced with magnetic stirring, and the reaction proceeded in the dark at 30 °C for a duration of 4 h (Scheme 1). To eliminate excess ascorbic acid and Na_2_SeO_3_, the solution underwent a 48 h dialysis process (MWCO 1000) using ultrapure water while being kept in the dark at 4 °C. After the dialysis procedure, the resulting solution was subjected to centrifugation at 15,000 rpm for 10 min. The resulting precipitate was carefully collected and subsequently freeze-dried, ultimately yielding the final product of TQ-SeNPs.

### 2.3 Characterization

The nanoparticles synthesized in this study, referred to as TQ-SeNPs, underwent comprehensive characterization using a variety of analytical techniques. An Agilent/Cary 60 spectrophotometer was used to collect UV–vis absorption spectra. Scanning electron microscopy (SEM) was also employed for the visual examination. Specifically, a drop of the sample was applied to a holey carbon film, and subsequent drying facilitated SEM imaging and energy-dispersive X-ray spectroscopy (EDS) mapping. These images and maps were acquired utilizing a microscope operating at 200 kV, produced by SU-1510, Hitachi High-Technologies Corp., Tokyo, Japan. Infrared absorption spectra of freeze-dried TQ-SeNP samples were captured across the 4000–400 cm^–1^ range using KBr pellets in conjunction with a Varian/660 IR spectrometer.

### 2.4 Cell Culture

The HEC1B cancer cell line was used in this study. HEC1B cells were grown in Dulbecco’s modified Eagle’s medium (DMEM) supplemented with 2 mM l-glutamine, penicillin (20 units/mL), streptomycin (20 μg/mL), and 10% heat-inactivated fetal bovine serum (FBS; Capricorn Scientific) at 37 °C in a saturated humidity atmosphere containing 5% CO_2_. HEC1B cells were seeded in 96-well plates at 5000 cells per well. After 24 h, cancer cells were treated with TQ-SeNP at different concentrations of 40, 80, 160, 320, and 640 μM.

### 2.5 Antiproliferative Activity

The antiproliferative effects of TQ-SeNPs on HEC1B endometrial cancer cells were determined using an XTT (2,3-bis (2-methoxy-4-nitro-5-sulfophenyl)-2*H*-tetrazolium-5-carboxanilide) assay at a concentration of 1 × 10^4^ cells per well in 96-well plates according to the kit’s instructions (Cell Proliferation Kit; Biological Industries Cat No: 20–300–1000). The XTT combination was administered in line with the dose and time prescribed by the manufacturer after the dosing intervals were completed. Formazan formation was measured spectrophotometrically with a microplate reader (Multiskan GO microplate spectrophotometer, Termo) at 450 wavelengths (reference wavelength 630 nm) and colorimetrically. The stated formula was used to calculate cell viability (percentage) using absorbance measurements.
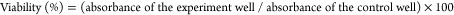


The AAT Bioquest online tool was used to assess the IC_50_ dosage of TQ-SeNPs on HEC1B cells (https://www.aatbio.com/tools/ic50-calculator). The IC_50_ dosage was used as a dose group in the other molecular studies of this study.

### 2.6 RNA Isolation, cDNA Synthesis, and RT-PCR Assay

Total RNA isolation from cells was carried out using Trizol (Invitrogen, USA) in accordance with the manufacturer’s instructions. The A.B.T. synthesis kit with RNase inhibitor was used for cDNA synthesis (A.B.T., Turkey). Real-time polymerase chain reaction (RT-PCR, Rotor Gene Qiagen, Germany) was used to visualize changes in ERK1, ERK2, MEK2 and NFKB (p65) mRNA expression. Normalization was accomplished through the use of beta-actin. The primer sequences used in this study are given in Table 1. SYBR Green qPCR Master Mix was used to perform RT-PCR assays using the ABT 2X qPCR SYBR-Green Master Mix protocol (Turkey).

**Table 1:** Primer Sequences of the Genes Were Used in the Study

gene name	forward	reverse
beta-actin	TCCTCCTGAGCGCAAGTACTC	CTGCTTGCTGATCCACATCTG
ERK1	TGGCAAGCACTACCTGGATCAG	GCAGAGACTGTAGGTAGTTTCGG
ERK2	ACACCAACCTCTCGTACATCGG	TGGCAGTAGGTCTGGTGCTCAA
MEK2	GTGGTCACCAAAGTCCAGCACA	CACGATGTACGGCGAGTTGCAT
NFKB (p65)	TGAACCGAAACTCTGGCAGCTG	CATCAGCTTGCGAAAAGGAGCC

### 2.7 ELISA Assay

The levels of phosphorylated-p38 mitogen-activated protein kinase (p38 MAPK) in the culture medium were evaluated using a p38 MAPK enzyme-linked immunosorbent assay kit (ELISA) (Lot no. 201910; Andy Gene, 301 University Village Drive, Richardson, TX75081, USA) in accordance with the manufacturer’s protocol.

### 2.8 Statistical Analysis

The parametric and nonparametric analyses of the dosage and control groups were performed using the IBM SPSS Version 23 (SPSS Inc., Chicago, IL, USA) analytical tool. In all statistical analyses, a p value less than 0.05 was accepted as statistically significant. In the analysis of RT-PCR data, quantitation was performed using the 2^–ΔΔ*CT*^ method via the RT-PCR analysis RT Profiler PCR Array Data Analysis program.

## 3 Results

### 3.1 Characterization of TQ-SeNPs

**Figure 1:**
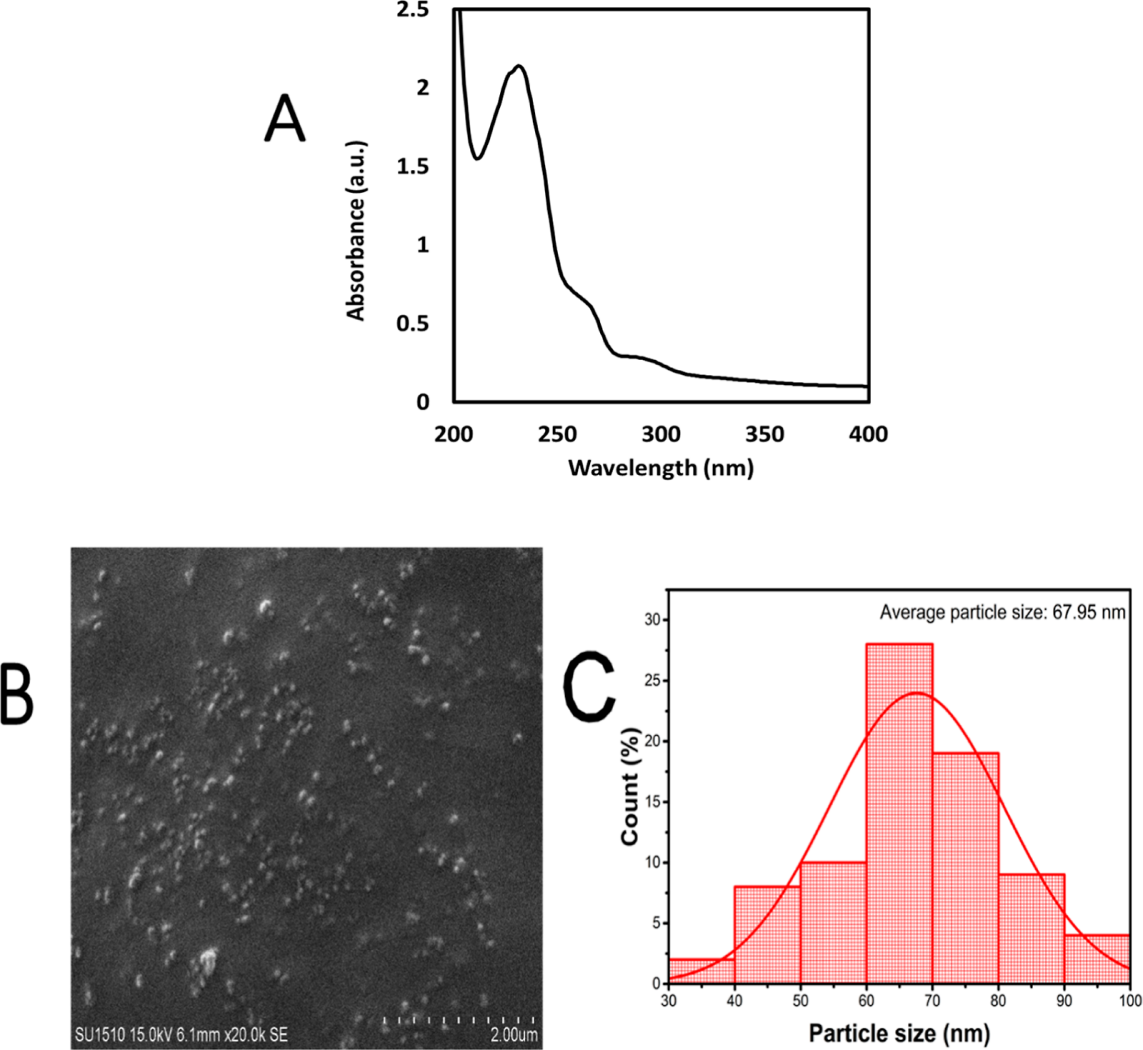
UV–vis spectrum of TQ-SeNPs (A), SEM image of TQ-SeNPs (B), and average particle size distribution graphic (C).

The absorption peak wavelengths can provide insights into the types of bonds present in a molecule and serve as a useful tool for identifying the functional groups within it. In the UV–vis spectrum of thymoquinone, a distinct absorption peak (λ_max_) is observed at 233 nm, as illustrated in Figure 1A. This particular peak is recognized as a characteristic feature of quinones, setting them apart from their analogue hydroquinone, which exhibits an absorption peak of around 290 nm.^27^ The impact of operational parameters on the yield of selenium nanoparticle (SeNP) synthesis was explored through UV–vis spectral analysis, as depicted in Figure 1A. This analysis revealed the emergence of SeNP formation within the wavelength ranges of 266–297 nm. For the investigation of the structure and dimensions of the synthesized SeNPs, the SEM image is presented in Figure 1B. This image provides visual confirmation of the creation of well-defined crystalline SeNPs exhibiting a spherical morphology, with sizes falling within the range of 30–90 nm. Furthermore, an observable delicate layer of biomolecules enveloping the SeNPs within the TQ solution is apparent. This biomolecular coating serves a dual purpose, acting as a barrier to impede the aggregation of SeNPs and contributing to their heightened stability. The particle size distribution of SeNPs is shown in Figure 1C, with the highest average intensity being at 68 nm.

**Figure 2:**
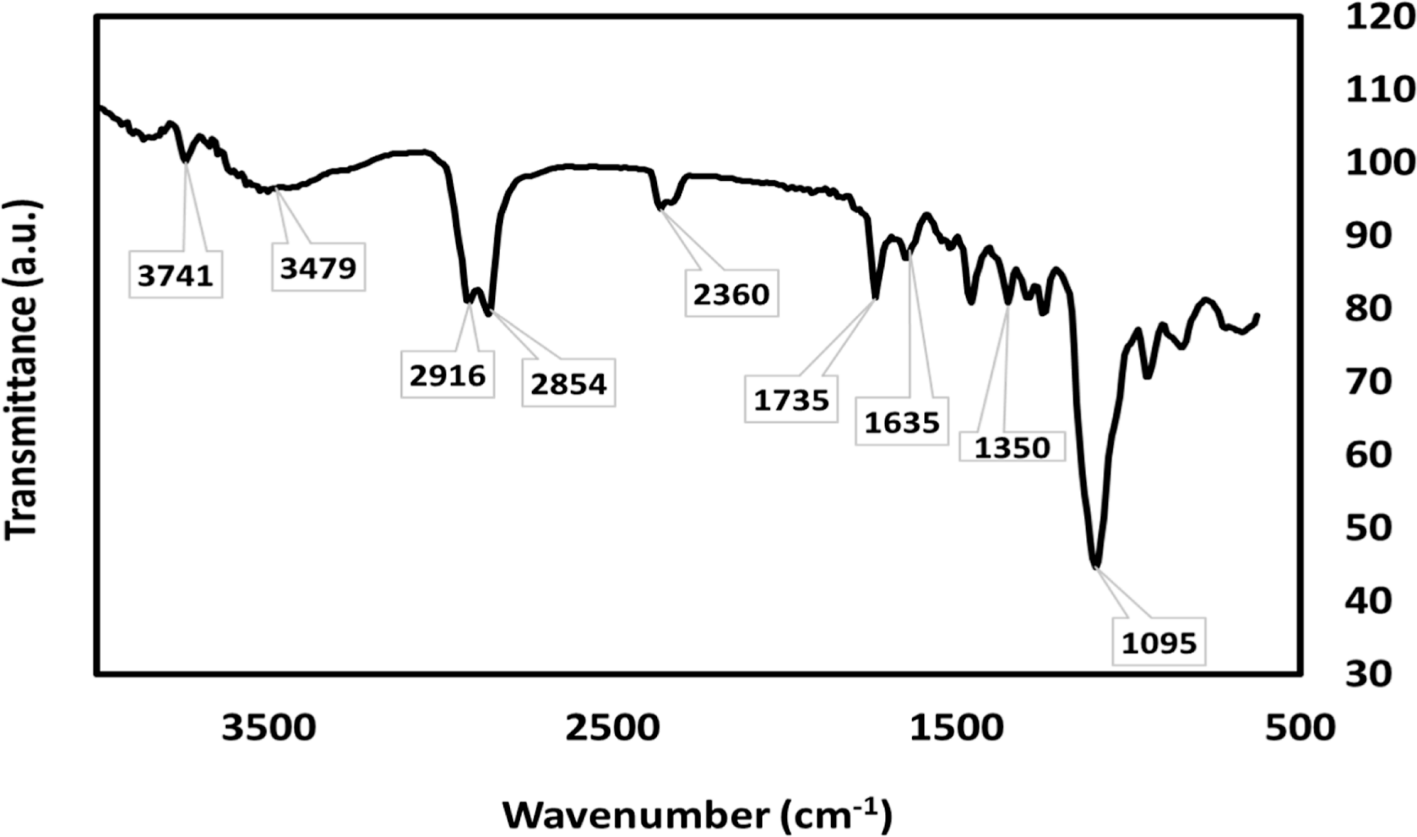
FT-IR results of TQ-SeNPs.

In Figure 2, the FT-IR spectrum of SeNPs synthesized under optimal conditions, utilizing ascorbic acid as a reducing agent and modified TQ as an anticancer agent, is displayed. A broad peak centered at 3479 cm^–1^ was identified, corresponding to the stretching vibrations of the O–H groups. Additionally, an absorption peak at 2916 cm^–1^ was linked to the C–H stretching of aromatic compounds present in the TQ compound. The absorption band at 1735 cm^–1^ is indicative of the presence of a carbonyl group (C=O) in the synthesized compound. The appearance of this band suggests a key modification of the molecular structure of TQ during the synthesis process. The notable band at 1625 cm^–1^ was attributed to the stretching vibrations of the C=C aromatic bond within phenolic groups, while the peak at 1350 cm^–1^ was associated with the bending vibrations of CH_2_ groups.

### 3.2 SEM Elemental Mapping and Composition

These findings provide confirmation of the presence of SeNP within the chemical composition of TQ. Furthermore, the existence of SeNPs was validated through distinctive stretching vibrations observed at 1095 cm^–1^ for terminal Se–O bonds and at 648 cm^–1^ for O–Se–O bonds.^28^

**Figure 3:**
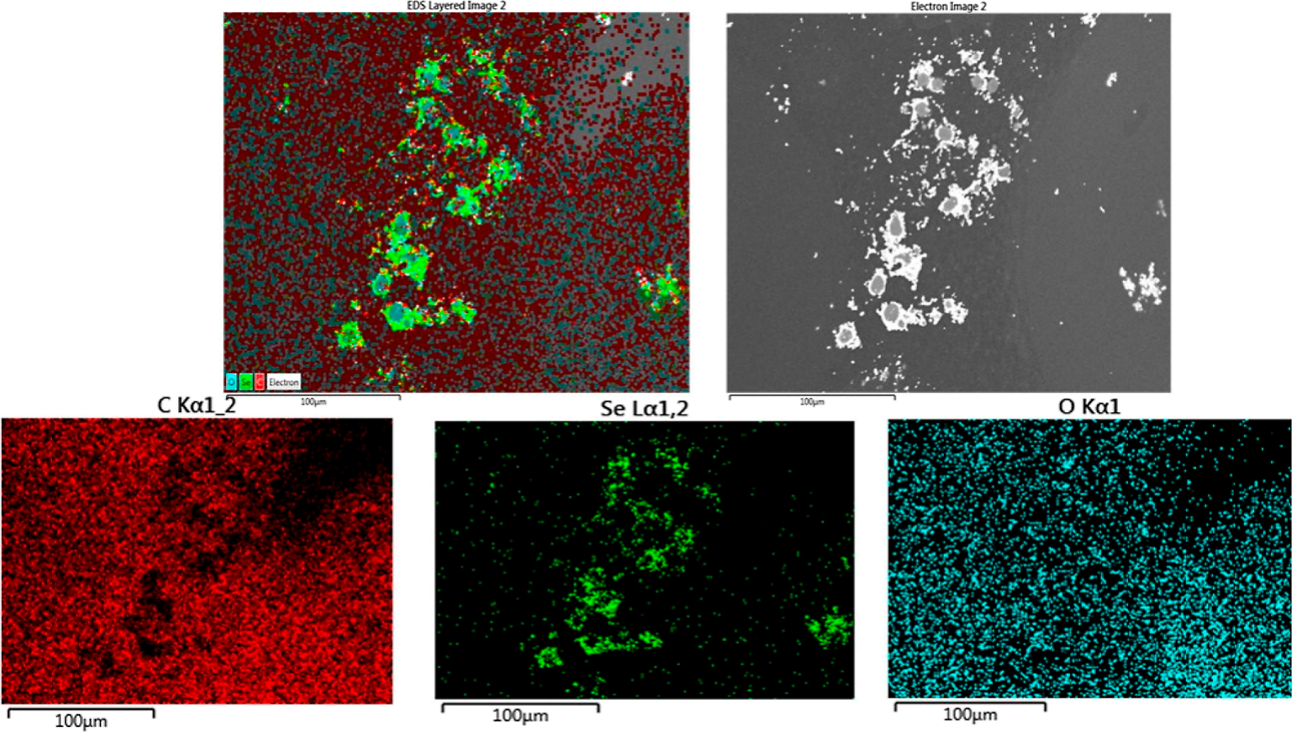
SEM mapping distribution of TQ-SeNPs.

The SEM mapping results in Figure 3 show that the region with SeNPs dripped on the surface is represented by an intense green color. The more dense carbon content due to the remaining carbon band of the sample dripped on the surface is shown in red. In addition, the atom of the TQ compound is shown in blue.

**Figure 4:**
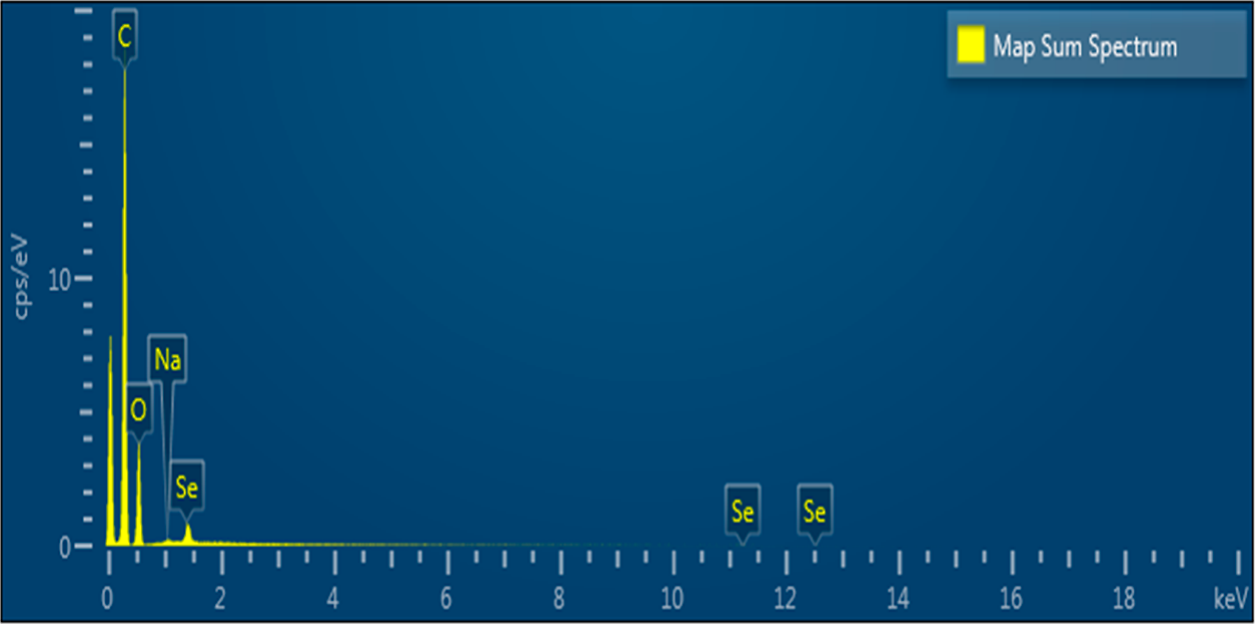
EDX analysis of TQ-SeNPs.

As demonstrated in Figures 4 and S1 (raw data) through the EDS analysis, the SeNP samples exhibited notable peaks, signifying the presence of Se, O, and C elements, each with substantial intensities. The emergence of signals corresponding to O and C can be attributed to the chemicals present in the TQ solution, which likely encapsulate the SeNP surface. Additionally, it is worth noting that the C and O peaks could also be influenced by the utilization of carbon tape as the substrate for the analysis. The quantification of elemental composition in the SeNP samples indicated mass percentages of 66.18% for C, 31.39% for O, and 2.23% for Se, respectively.

### 3.3 Antiproliferative Activity

**Figure 5:**
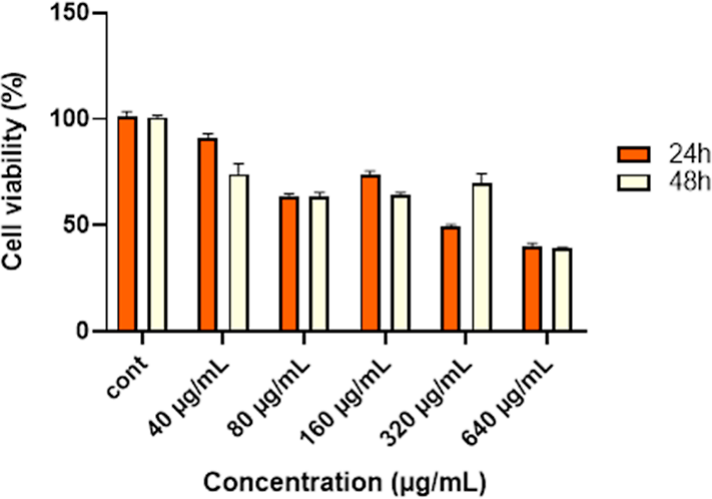
HEC1B cells were treated with TQ-SeNP at 40–640 μg/mL concentrations and time intervals, and their viability was assessed by XTT assay. IC_50_ doses of TQ-SeNP in HEC1B cells were detected as 526.45 μg/mL at the 48th hour.

The effects of TQ-SeNP on cell viability in HEC1B endometrial cancer cells were measured in a time- and dose-dependent manner using the XTT colorimetric cytotoxicity test. In cells treated with TQ-SeNP at different concentrations (40–640 μg/mL), the IC_50_ dose was found to be 526.45 μg/mL at the 48th hour (Figure 5).

### 3.4 Real-Time-PCR Results

The expression analysis of ERK1, ERK2, MEK2, and NFKB (p65) was performed by RT-PCR. As a result of RT-PCR, mRNA expression changes of genes involved in the MAPK signaling pathway were evaluated. All gene fold changes and *p* values are summarized in Table 2.

**Table 2:** Fold Regulation and *p* Values in TQ-SeNP-Treated Dose Groups Compared with Those in the Control Group (*p* < 0.05)

gene names	fold regulation	*p* value
ERK1	2.33	*0.475408*
ERK2	–2.3	*0.151184*
MEK2	–1.65	*0.381820*
NFKB (P65)	–4.32	*0.163245*

When the dose group treated with 526.45 μg/mL TQ-SeNP at 48 h was compared with the control group, the down-regulation of ERK2 (−2.3, *p* = 0.151184), MEK2 (−1.65, *p* = 0.381820), and NFKB (−4.32, *p* = 0.163245) gene expression in the dose group was not significant (*p* > 0.05). Although there was an increase in the level of ERK1 (2.33, *p* = 0.475408) mRNA expression, it was still not statistically significant (Table 2).

### 3.5 ELISA Assay

**Figure 6:**
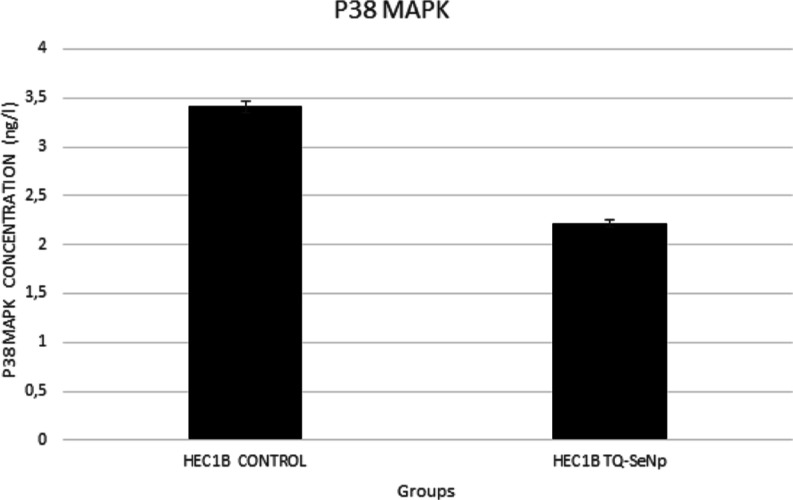
ELISA decreased the concentration of p38 MAPK in HEC1B cells treated with the TQ-SeNP treatment dose.

ELISA showed that a dose of 526.45 μg/mL administered at the 48th hour of the TQ-SeNP treatment reduced the p38 MAPK concentration (2.2169 ng/L) (Figure 6).

## 4 Discussion

MAPK pathways are a network of structures that regulate many physiological processes, especially cell growth, differentiation, and apoptotic cell death.^29^ MAPK signaling pathway activation changes or mutations are known to exist in EC and different types of cancer.^30−32^ Nanoparticle drug designs based on target genes or proteins that minimize toxicity in cancer treatments have taken an important place. Therefore, in this study, TQ was encapsulated into Se nanoparticles for the first time using a nanotechnological approach to improve the delivery of TQ in cells and limit its cytotoxicity as TQ is a promising anticancer molecule candidate. And in this study, we aimed to see possible increases in the ability of TQ-SeNP to target HEC1B EC cells at the molecular level.

TQ is a well-known natural compound that has been frequently studied for the treatment of various types of cancer. TQ is thought to exert its anticancer properties by interfering with various oncogenic pathways. It is known to prevent inflammation and oxidative stress,^16,33^ inhibit angiogenesis and metastasis,^34^ induce apoptosis,^35,36^ and regulate up- and down-regulation of various genes such as tumor suppressors.^37^

It is known that ongoing inflammation causes the cell to progress in the oncogenic direction. Cyclooxygenase-2 (COX-2), arachidonate 5-lipoxygenase (5-LOX), inducible nitric oxide synthase (iNOS), and A2-phospholipase are inflammatory mediators. These mediators have been reported to play a role in the carcinogenic process. TQ exerted its anti-inflammatory activity by inhibiting the iNOS pathway and downregulating COX-2 expression.^16^

The most prominent feature of cancer cells is that they continue to divide without stopping. The division-suppressing property of TQ continues to guide studies. TQ exerts its anticancer effect by stopping the cell cycle and disrupting microtubule structuring. In breast and colon cancers, it suppresses cyclins D and E in the G1 phase and upregulates p21 and p27. This leads to cell cycle arrest.^33^ TQ binds to the colchicine binding site, degrades alpha and beta microtubules, and inhibits microtubule remodeling.^16^

Apoptosis is controlled by maintaining a balance between proapoptotic proteins such as Bax and Bak and antiapoptotic proteins such as Bcl-2 and Bcl-XL in the mitochondrial membrane. In the presence of DNA damage, cytochrome C is released outside the cell, caspase-9 is activated, and DNA is fragmented. The apoptotic effect of TQ has been reported in squamous cell carcinoma.^36^

TQ has also been shown to inhibit invasion and migration. It shows this effect by promoting the production of matrix metalloproteinases (MMPs). It has been shown to downregulate MMP-2 and MMP-9 in glioblastoma cells.^35^ PTEN is a tumor suppressor gene, and mutations of the PTEN gene have been detected in various cancers. TQ exerts its effect by upregulating the PTEN gene, stopping the cell cycle, and activating apoptosis, thereby inhibiting cell proliferation.^37^

Although TQ is a biomolecule with promising anticancer activity, its bioavailability is low.^10,20,21^ Therefore, TQ is encapsulated with various nanoparticles to increase its absorption by tumor cells.^10^

Muizzuddin Bin Mohd Rosli et al. encapsulated TQ with PLGA (polylactic-*co*-glycolic acid) and examined the nanoparticle effect in the malignant melanoma cancer cell line. At the 24th hour, they showed that it caused higher toxicity in tumor cells.^38^ In another study, they coated TQ with a zinc oxide nanoparticle in the MDA-MB231 cell line, examined its effect on breast cancer cells, and showed by the flow cytometric method that it stopped DNA damage and cell cycle and increased apoptosis.^39^ Ibiyeye et al. coated TQ with a CaCO_3_ nanoparticle and investigated its antiproliferative effect on the same tumor cell line (MDA-MB231 cell line) and found that the IC_50_ value of the TQ-CaCO_3_ nanoparticle was higher than that of TQ. However, the combination therapy showed improved apoptosis, reduced intracellular migration, and reduced invasion when compared with single drug-loaded nanoparticles and free drugs.^40^ TQ is also coated with myristic acid-chitosan^41^ and paclitaxel.^42^ The antiproliferative effect of these nanoparticles was measured in MCF7 cancer cells by an MTT assay. In both studies, the anticancer activity of encapsulated nanoparticles was higher than that of other free drugs.^42^

Nanoformulation studies have begun to take an important place in the literature in order to increase the effect of a drug with a low therapeutic bioavailability. El-Ashmawy and colleagues studied doxorubicin (DOX).^43^ Although DOX is an effective anticancer drug, its cardiotoxic effects are quite high. In this study, DOX and TQ were loaded into the F2 gel (fully acetylated poly-*N*-acetyl glucosamine nanofiber). The combination of DOX and TQ showed significant tumor reductions. This decrease was caused by upregulating p53, downregulating Bcl-2, and inducing apoptosis. In the same study, it was also determined that the nanoforms of the DOX-TQ combination accelerated drug distribution. Topotecan is another drug that is used to treat cancer. It causes DNA damage in the cell by inhibiting the DNA topoisomerase-I enzyme. Topotecan-TQ nanoforms increased anticancer activity, especially in ovarian and lung tumors.^44^ Amphotericin-B is a drug with antifungal effects. It has been shown that the nanoparticle forms of TQ are more effective on Candida yeasts than the free forms of the drug.^45^ Another study was conducted by studying the effects of the nanoforms of TQ and metformin in diabetic rats.^46^ In this study, TQ and metformin-loaded nanoparticles showed better release than their free forms, and a significant decrease in the serum lipid and blood sugar levels of diabetic rats was observed.

Nanoparticle forms of drugs have started to be preferred more than traditional drug forms because they offer improved safety. In these type of drugs, mainly Se, Ag, Au, and Zn metals and chitosan are used, with the size of 12–140 nm,^47^ an average of 80 nm,^48^ of 84 nm,^49^ an average of 8 nm,^50^ and an average of 60 nm, respectively.^51^

We see selenium (Se) in human metabolism as selenocysteine in various antioxidant enzymes such as glutathione peroxidase and thioredoxin reductase. Se is necessary for the biochemical activities of these enzymes. SeNPs are highly attractive due to their anticancer activity and reduced toxicity.^52^ While it has minimal side effects in normal cells, it has been reported to induce apoptosis in cancer cells by the mitochondrial pathway at doses close to toxic levels.^53−56^ Se doses reduce the incidence of many cancers, including lung, prostate, and colorectal cancer.^57^ Therefore, the pharmacological action and toxicity are critically dependent on the concentration and type of Se compound used.^52^

The study has some limitations. The first reason is that the expression changes of most of the MAPK signaling pathway genes were not examined. Second, more toxicity studies are needed to demonstrate the in vivo efficacy of nanoparticles through animal modeling and clinical studies.

## 5 Conclusions

This research focused on investigating the potential anticancer properties of TQ-SeNPs in HEC1B endometrial carcinoma cells. This study involved the synthesis and comprehensive characterization of TQ-SeNPs, including their size, morphology, and elemental composition. Morphological changes induced by these nanoparticles were examined using SEM.

The results demonstrated a notable decrease in the viability of HEC1B endometrial carcinoma cells in a time- and dose-dependent manner upon treatment with TQ-SeNPs. The calculated IC_50_ dose of TQ-SeNPs at the 48th h time point was determined to be 526.45 μg/mL. Additionally, ELISA analysis revealed that TQ-SeNP treatment led to a reduction in the levels of p38 MAPK, suggesting a potential involvement of the MAPK signaling pathway in the observed anticancer effects. The initial mechanistic study on the efficacy of TQ-SeNPs in EC under in vitro conditions has implicated the MAPK signaling pathway. The obtained results will shed light on more detailed molecular biology investigations to be conducted in the future. Demonstrating the antiproliferative activity of TQ-SeNPs in other EC cell lines in vitro and conducting in vivo animal experiments will contribute to this field in the future.

Therefore, we encapsulated TQ into Se nanoparticles for the first time using a nanotechnology approach to improve the delivery of TQ in cells and limit unwanted cytotoxicity, and in this study, we aimed to see possible increases in the ability of TQ-SeNP to target HEC1B EC cells at the molecular level.

## Data Availability

The data sets generated and analyzed during the current study are available from the authors on reasonable request.

## Supporting Information Available

Raw data of the EDS spectrum (PDF)

## Notes

Ethical Approval: Since it is a cell culture study, it does not require ethics committee approval.
