# Supplementary material for: Exploring the Potential of Thymoquinone-Stabilized Selenium Nanoparticles: In HEC1B Endometrial Cancer Cells Revealing Enhanced Anticancer Efficacy
Source: ACS Omega. 2023 Oct 16;8(42):39822–9. doi: 10.1021/acsomega.3c06028 (PMC10601430; doi:10.1021/acsomega.3c06028)
Supplement: Supplementary file 1 — ao3c06028_si_001.pdf [file ao3c06028_si_001.pdf]

Exploring the Potential of Thymoquinone Stabilized Selenium Nanoparticles: In HEC1B Endometrial Cancer Cells Revealing Enhanced Anticancer Efficiacy

Gonca Gulbay[1], Mucahit Secme[1], Hasan Ilhan[2*]

[1]Department of Medical Biology, Faculty of Medicine,
Ordu University, Ordu, Turkey
[2] Department of Chemistry, Faculty of Science,
Ordu University, Ordu, Turkey

Dr. Hasan Ilhan (PhD)
(Corresponding Author)
Department of Chemistry, Faculty of Science,
Ordu University, Ordu, Turkey
E-mail address: hasanilhan@odu.edu.tr
fax no: 0 452 226 52 28
Zip code:52200
Telephone number: 0542 319 18 07
ORCID ID. 0000-0002-4475-1629

**Map Sum Spectrum**				
**Element**	**Line Type**	**Weight %**	**Weight % Sigma**	**Atomic %**
C	K series	66.18	0.43	73.38
O	K series	31.39	0.43	26.13
Se	L series	2.23	0.12	0.38
Na	K series	0.20	0.06	0.12
Total		100.00		100.00

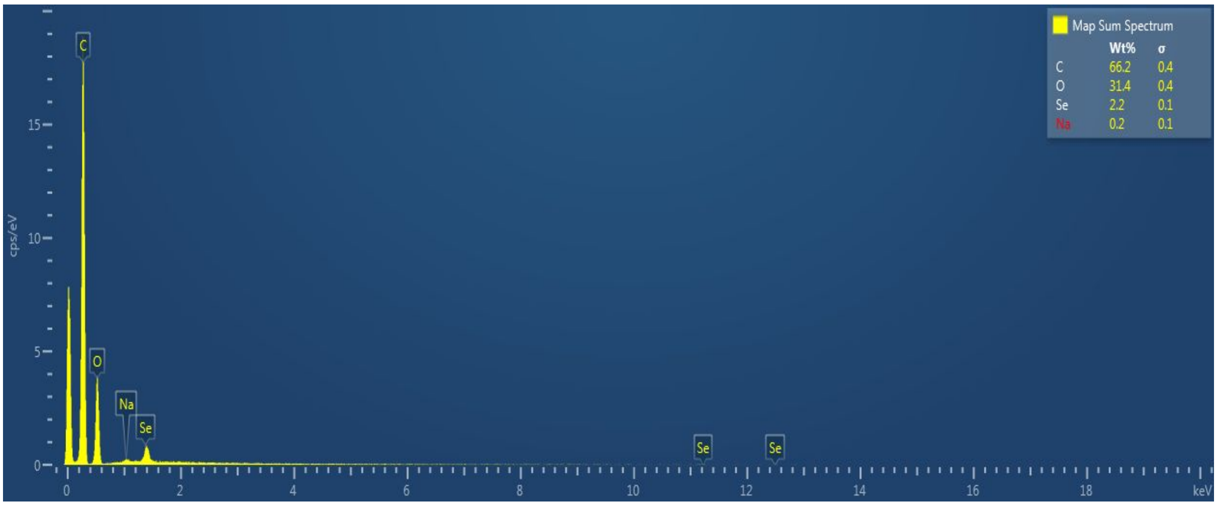

Figure S1. The Raw data of Energy Dispersive X-ray Spectroscopy (EDS) spectrum.
